# Transcriptomic Changes in Liver of Young Bulls Caused by Diets Low in Mineral and Protein Contents and Supplemented with n-3 Fatty Acids and Conjugated Linoleic Acid

**DOI:** 10.1371/journal.pone.0167747

**Published:** 2016-12-08

**Authors:** Sara Pegolo, Alessio Cecchinato, Núria Mach, Massimiliano Babbucci, Marianna Pauletto, Luca Bargelloni, Stefano Schiavon, Giovanni Bittante

**Affiliations:** 1 Department of Agronomy, Food, Natural Resources, Animals and Environment (DAFNAE), University of Padova, Legnaro, Padova, Italy; 2 Animal Genetics and Integrative Biology unit (GABI), INRA, AgroParisTech, Université Paris-Saclay, Jouy-en-Josas, France; 3 Department of Comparative Biomedicine and Food Science, University of Padova, Legnaro, Padova, Italy; Cornell University, UNITED STATES

## Abstract

The aim of the present study was to identify transcriptional modifications and regulatory networks accounting for physiological and metabolic responses to specific nutrients in the liver of young Belgian Blue × Holstein bulls using RNA-sequencing. A larger trial has been carried out in which animals were fed with different diets: 1] a conventional diet; 2] a low-protein/low-mineral diet (low-impact diet) and 3] a diet enriched in n-3 fatty acids (FAs), conjugated linoleic acid (CLA) and vitamin E (nutraceutical diet). The initial hypothesis was that the administration of low-impact and nutraceutical diets might influence the transcriptional profiles in bovine liver and the resultant nutrient fluxes, which are essential for optimal liver function and nutrient interconversion. Results showed that the nutraceutical diet significantly reduced subcutaneous fat covering *in vivo* and liver pH. Dietary treatments did not affect overall liver fat content, but significantly modified the liver profile of 33 FA traits (out of the total 89 identified by gas-chromatography). In bulls fed nutraceutical diet, the percentage of n-3 and CLA FAs increased around 2.5-fold compared with the other diets, whereas the ratio of n6/n3 decreased 2.5-fold. Liver transcriptomic analyses revealed a total of 198 differentially expressed genes (DEGs) when comparing low-impact, nutraceutical and conventional diets, with the nutraceutical diet showing the greatest effects on liver transcriptome. Functional analyses using ClueGo and Ingenuity Pathway Analysis evidenced that DEGs in bovine liver were variously involved in energy reserve metabolic process, glutathione metabolism, and carbohydrate and lipid metabolism. Modifications in feeding strategies affected key transcription factors regulating the expression of several genes involved in fatty acid metabolism, e.g. insulin-induced gene 1, insulin receptor substrate 2, and RAR-related orphan receptor C. This study provides noteworthy insights into the molecular changes occurring as a result of nutrient variation in diets (aimed at reducing the environmental impact and improving human health) and broadens our understanding of the relationship between nutrients variation and phenotypic effects.

## Introduction

Growing attention has been recently paid to sustainability in the livestock sector, the functionality of animals, and product quality according to the EU Framework Program Horizon 2020 [1]. Sustainable beef production depends on a balanced combination of various factors, such as the reduction of negative environmental impacts, animal health, nutritional efficiency, efficient growth, and a desirable carcass composition with a high percentage of lean tissue and a low fat content. The Nitrate Directive (91/676/EC), which regards the protection of water quality across Europe, together with the increasing cost of protein sources have brought pressure on farmers to reduce the N content in livestock diets thereby reducing N excretion and emission [2–3]. Minerals, which are often given to animals in excess of their requirements [4], can also contribute to environmental pollution (especially P, Cu and Zn).

Aside from environmental issues, a growing awareness of the association between diet and human health has increased the demand for foods of high nutritional quality. There is now recognition of the importance of fatty acids (FAs) of the n-3 series and conjugated linoleic acid (CLA), the predominant isomers being 18:2*c*9,*t*11 (9-CLA, the rumenic acid, RA) and 18:2*t*10,*c*12 (10-CLA), for their potential beneficial effects on the functioning of the immune, nervous, and cardiovascular systems in humans [5], as well as for reproductive performances and carcass traits in ruminants [6]. Dietary supplementation with n-3 FAs or CLA has been found to affect FA composition in ruminant tissues [7–9]. Specific effects have been ascribed to CLA mixtures, where two isomers generally dominate, i.e. 18:2*c9*,*t11* and 18:2*t*10,*c*12. The former isomer has anti-carcinogenic properties [10], whereas the latter can increase the lean to fat ratio in growing animals [11–12]. Adaptation to low protein and minerals, and to high n-3 FAs and CLA ingestion is influenced by the transcriptional and translational regulation of genes that encode the proteins controlling these processes. To our knowledge, studies are available about the effects of these FAs on the expression of target genes in ruminants [13–15] but no information is available on the effects of protein and mineral deficiency.

Transcriptomic approaches have been applied for genome-wide screening in blood and tissue biopsies aimed at better understanding the gene expression networks that control nutrient utilization. In beef cattle, few studies adopted RNA-sequencing methodology aiming to improve the understanding of the supplement-driven physiological and metabolic mechanisms that regulate the global tissue response to nutrients [16–18].

In ruminants, *de novo* fatty acid synthesis predominantly takes place in the adipose tissue but also the liver has a specific role in lipid metabolism [19]. In particular, ruminant liver is involved in the uptake, oxidation and metabolism of non-esterified fatty acids (NEFAs), synthesis of cholesterol and phospholipids, and synthesis and secretion of lipoproteins. Polyunsaturated fatty acids (PUFAs), after rumen biohydrogenation, could be further metabolized in the liver and their subsequent deposition in muscle may be affected [20]. Thereby, modification of muscle fatty acid composition aiming to enhance the nutritional value of meat should take into consideration liver metabolism. We hypothesized that identification of the liver genes specifically regulated by n-3 and CLA, as well as the variations in dietary protein and mineral contents could reveal unique biomarkers of physiological adaptation to diet supplementation and could also provide significant insights into the molecular control of this response and the resulting phenotypes. Based on the phenotypic results of a large experimental trial, involving males and females of Belgian Blue × Holstein and Belgian Blue × Brown Swiss, we selected the 12 Belgian Blue × Holstein bulls (because of their greater importance for the beef industry in Europe) to perform a comparative transcriptome analysis of liver in bulls fed with a conventional diet (Conv), a low-protein/low-mineral diet (low-impact diet, LowI) or a low-protein/low-mineral diet supplemented with n-3 FAs, CLA, and vitamin E (nutraceutical diet, Nutr) using RNA-sequencing. The specific aims of this work were: (i) to describe the changes in liver gene expression profiles and phenotypic variations when comparing a Conv with a LowI or a Nutr diet, (ii) to identify the biological processes in liver regulated by variations in diet composition, iii) to define the relationships between the expression profiles of regulated genes and phenotypic traits such as FA profiles.

## Results

### Phenotypic analyses

#### Growth performances, carcass and liver traits

No significant differences among treatments were found for body weight (BW), average daily gain (ADG), and *in vivo* muscularity score (Table 1).

**Table 1:** Growth performances, carcass traits, liver characteristics of young bulls fed conventional, low-impact and nutraceutical diets.

	Diet^1^				
	Conv	LowI	Nutr	SEM	P- values^2^
**Growth performances**					
Final body weight, kg	684	671	662	17.23	0.893
Average daily gain, kg	1.54	1.42	1.38	0.03	0.366
Muscularity score^3^	4.22	4.17	4.25	0.07	0.916
Fatness score^3^	1.89^a^	1.83^a^	1.41^b^	0.06	0.042
**Carcass traits**					
Carcass weight, kg	396	388	382	9.49	0.580
Carcass yield, g/kg	0.580	0.578	0.576	0.002	0.619
Muscularity score^4^	4.00	4.00	3.75	0.12	0.788
Fatness score^4^	2.00	2.08	1.83	0.11	0.641
**Liver**					
Weight, kg	6.53	6.36	6.90	0.15	0.750
Dry matter, %	29.23	29.61	29.45	0.15	0.515
Fat, %	3.06	2.45	2.38	0.22	0.358
Protein, %	19.85	19.23	19.67	0.12	0.149
Ash, %	1.53	2.09	1.77	0.07	0.066
pH	6.13^a^	6.14^a^	6.08^b^	0.01	0.044

SEM: Mean standard error of the means.

^1^Mean values are reported for each diet.

^2^*P*-values after Kruskal Wallis non-parametric test

^3^In accordance with Schiavon et al. [21], body conformation was linearly scored in vivo from S+ (all muscle profiles extremely convex; exceptional muscle development) to P- (all muscle profiles concave to very concave; poor muscle development) taking into account the profiles of shoulders, loins, rump, thighs, and buttocks (S+ = 6.33; P- = 0.66), and fat covering was linearly scored *in vivo* in 5 classes (1 = very lean; 5 = very fat) using a combined visual and palpation approach taking into account the presence and thickness of subcutaneous fat depots at the base of the tail, ribs, and shoulders.

^4^SEUROP scoring system for carcass muscularity from S+ (all muscle profiles extremely convex; exceptional muscle development) to P- (all muscle profiles concave to very concave; poor muscle development) taking into account the profiles of shoulders, loins, rump, thighs, and buttocks (S+ = 6.33; P- = 0.66), and for carcass fat covering (1 = very lean; 5 = very fat).

Letters indicate significant differences (*P* < 0.05; Bonferroni post-hoc test).

In contrast, significantly lower values of *in vivo* fat covering were found with Nutr compared with Conv and LowI groups (*P <* 0.05). None of the carcass traits considered (carcass weight and yield, muscularity and fatness scores) differed significantly among treatments, even though muscularity and fatness scores were nominally lower in Nutr compared with Conv and LowI treatments. No differences in liver weight among treatments were observed. Average liver pH was significantly lower when animals were fed the Nutr diet than when fed the LowI or Conv diet.

#### Liver fatty acid and mineral profile

Nineteen FAs out of the total 89 identified by GC showed significant differences (*P <* 0.05) among dietary treatments (Table 2).

**Table 2:** Liver fatty acid (FA) contents^1^ of young bulls fed conventional (Conv), low-impact (LowI) and nutraceutical (Nutr) diets.

	Diet^2^				
	Conv	LowI	Nutr^3^	SEM	*P*-values^4^
**Individual FAs, g/100g FAs**					
17:0*iso*	0.336^a^	0.291^ab^	0.249^b^	0.006	0.034
17:0*anteiso*	0.696	0.481	0.400	0.016	0.051
24:0	0.215^ab^	0.147^b^	0.278^a^	0.009	0.034
14:1 others^5^	0.023^ab^	0.034^a^	0.004^b^	0.002	0.042
15:1 sum^5^	0.006	0.024	0.000	0.001	0.058
17:1 sum^5^	0.261	0.219	0.158	0.006	0.051
18:1*t*12	0.451	0.677	0.721	0.017	0.055
18:1*c*9	8.887	8.208	6.348	0.209	0.050
18:1*c*11	1.025^a^	0.919^ab^	0.682^b^	0.011	0.034
18:1*c*12	0.349^b^	0.501^ab^	0.585^a^	0.012	0.043
18:1 others^6^	0.400^b^	0.682^ab^	1.308^a^	0.028	0.018
18:2*c*9,*c*12	15.932^b^	20.181^a^	16.129^b^	0.160	0.038
18:3*c*9,*c*12,*c*15	0.620^b^	0.759^ab^	2.263^a^	0.033	0.034
18:3 others^5^	0.003^b^	0.000^b^	0.620^a^	0.061	0.022
18:2*c*11,*t*13 (CLA)	0.028^b^	0.034^b^	0.129^a^	0.006	0.034
18:2*c*9,*c*11 (CLA)	0.014^b^	0.013^b^	0.073^a^	0.008	0.030
18:2*t*10,*c*12 (CLA)	0.000^b^	0.016^b^	0.132^a^	0.005	0.028
20:3*c*8,*c*11,*c*14	4.784	2.740	3.104	0.147	0.064
20:4*c*8,*c*11,*c*14,*c*17	0.315^a^	0.212^a^	0.882^ab^	0.014	0.043
20:4 others^5^	0.120	0.116	0.255	0.004	0.055
20:5*c*5,*c*8,*c*11,*c*14,*c*17 (EPA)	0.332^ab^	0.245^b^	0.920^a^	0.011	0.018
22:4*c*7,*c*10,*c*13,*c*16	3.753^a^	2.744^a^	1.995^ab^	0.083	0.018
22:5*c*4,*c*7,*c*10,*c*13,*c*16	0.884	0.610	0.613	0.019	0.055
22:5*c*4,*c*7,*c*10,*c*13,*c*16,*c*19 (DPA)	1.929^ab^	1.445^b^	3.734^a^	0.033	0.018
20:2 to 20:5 others^5^	0.065	0.017	0.082	0.006	0.065
22:6*c*4,*c*7,*c*10,*c*13,*c*16,*c*19 (DHA)	0.322	0.292	0.617	0.016	0.050
**Groups of FA, g/100g FAs**					
PUFA	40.767	40.832	44.142	0.256	0.057
CLA *c*,*t*/*t*,*c*	0.365	0.409	0.894	0.481	0.050
CLA total	0.445	0.474	1.053	0.024	0.050
*Trans* 18:1	1.623^b^	2.418^ab^	2.896^a^	0.078	0.043
n-6	35.296	35.477	32.031	0.253	0.055
n-3	3.995^ab^	3.923^b^	9.696^a^	0.116	0.043
n-6/n-3	8.855^ab^	9.257^a^	3.327^b^	0.124	0.043

SEM: Mean standard error of the means; CLA: conjugated linoleic acid; PUFA: polyunsaturated fatty acids; EPA: eicosapentaenoic acid; DPA: docopentaenoic acid; DHA: docohexaenoic acid

Letters indicate significant differences (*P* < 0.05; Bonferroni post-hoc test)

^1^Only the traits with significant differences (*P* < 0.05) or trends (*P* < 0.10) are reported

^2^Mean values are reported for each diet.

^3^One animal was not included in the FA analysis due to bad quality of the chromatography data.

^4^*P*-values after Kruskal Wallis non-parametric test.

^5^Sum of positional isomers

^6^18:1 others: sum of 18:1*t*4, 18:1 *t*6, 18:1*t*8, 18:1*t*9, 18:1*t*10, 18:1*t*13 + *t*14, 18:1*c*13, 18:1*t*16, 18:1*c*14, 18:1 *c*15, and 18:1*c*16.

Only some minor individual saturated FAs were affected (*P* < 0.05) by dietary treatments (17:0*iso* and 24:0). The proportions of some monounsaturated FAs (MUFAs) were affected by the Nutr diet. In particular, oleic acid (18:1*c9*) was lower in Nutr young bulls compared with Conv (-29%) and LowI (-23%) treatments (*P* = 0.050). A notable reduction in 18:1*c11*, and some medium-chain MUFAs (14:1 isomers) was also observed in Nutr *vs* Conv (*P* = 0.042 and *P* = 0.034, respectively), while increased proportions of 18:1*c12 (P* = 0.034*)* and other 18:1 isomers were detected (*P* = 0.018).

The relative content of some PUFAs was affected by the Nutr diet (Table 2). For instance, significant differences (*P* = 0.038) were detected for the linoleic acid (18:2 *c*9,*c*12) content, which represented almost half of all PUFAs in the liver of LowI bulls, and only 39% of Conv and 37% of Conv bulls. Compared with Conv, Nutr strongly increased the content of alpha-linolenic acid (18:3*c*9,*c*12,*c*15; +365%) and other minor 18:3 isomers (*P*<0.05). The CLA *c*,*t*/*t*,*c* isoforms and total CLA isomers increased about 2.5-fold in Nutr compared with Conv and LowI (*P* = 0.050). About individual CLA contents, large differences were found for 18:2*c*9,*c*11, 18:2*t*10,*c*12 and 18:2*c*11,*t*13 (*P*<0.05). The n-3 FAs proportion also increased 2.5-fold in Nutr, with a contemporaneous reduction in the n-6/n-3 ratio in Nutr compared with the other groups (*P* = 0.043). In addition, n-3 FAs eicosapentaenoic acid (EPA) and docopentaenoic acid (DPA) were much higher (*P* = 0.018) in Nutr compared with Conv and LowI. *Trans* 18:1 FAs significantly increased in the young bulls fed Nutr (1.8-fold, *P* = 0.043) compared with Conv.

The content of 27 minerals in liver was determined by inductive couple plasma-optical emission spectroscopy (ICP-OES), and of these 27 minerals, 11 (As, B, Ba, Co, Cr, Hg, Li, Ni, PB, Sb, and V) were below the limit of detection. Aluminum and Si were excluded due to a number of missing data. Liver concentrations were detected for 14 minerals: Ca, Cd, Cu, Fe, K, Mg, Mn, Mo, Na, P, S, Se, Sn, and Zn (Table 3), but no significant differences were observed among the 3 feeding groups.

**Table 3:** Mineral contents of diets and liver of young bulls fed conventional (Conv, n = 3), low-impact (LowI, n = 4) and nutraceutical diets (Nutr, n = 4).

	Diet^1^				
	Conv	LowI	Nutr	SEM^2^	*P*-values^2^
**Micro-element, mg/kg**					
Cd	0.065	0.070	0.066	0.003	0.47
Cu	91.2	60.1	66.4	8.51	0.11
Fe	41.1	45.2	36.9	3.29	0.19
Mn	2.64	2.26	2.51	0.10	0.14
Mo	1.16	1.06	1.11	0.07	0.65
Se	0.48	0.42	0.50	0.02	0.16
Sn	0.73	0.78	0.66	0.05	0.33
Zn	33.2	25.6	27.9	2.11	0.12
**Macro-element, mg/kg**					
Mg	131	128	131	3.8	0.59
Ca	40	41	37	1.7	0.30
K	2450	2482	2533	74.6	0.70
Na	684	707	721	18.7	0.46
P	2655	2693	2692	72.0	0.74
S	1555	1506	1561	31.9	0.33

SEM: Mean standard error of the means.

^1^Mean values are reported for each diet.

^*2*^*P*-values after Kruskal Wallis non-parametric test.

### Transcriptomic data analyses

#### Sequencing and mapping

**Fig 1:**
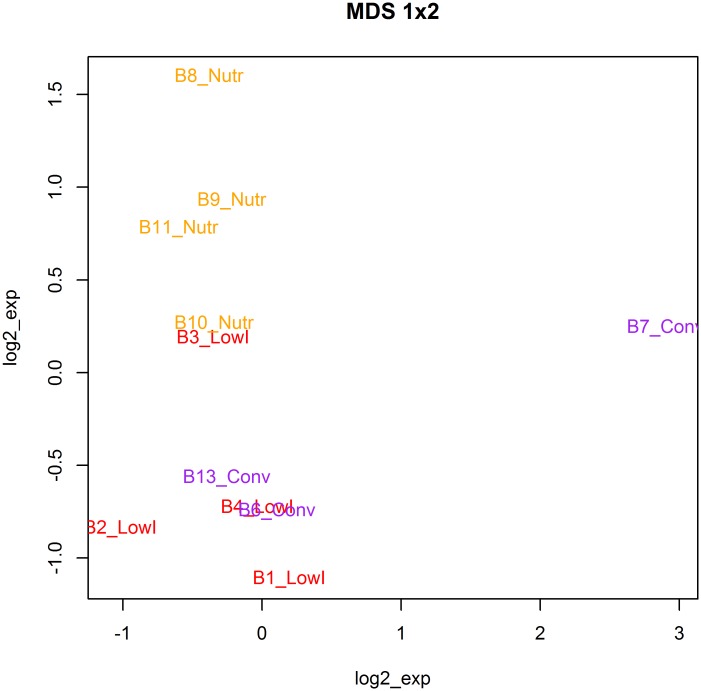
Multidimensional scaling (MDS) analysis using log2 of all normalized unique gene counts (n = 24,596). Conv: conventional diet; LowI: low-impact diet; Nutr: nutraceutical diet.

RNA-sequencing data were obtained from 3 Conv bulls, 4 LowI bulls and 4 Nutr bulls. All samples passed quality control measures for raw and trimmed sequenced reads. An average of over 20 million reads were obtained per sample, with ~80% of the reads mapping to the bovine reference genome (see summary statistics in S1 Table). High correlations between read count tools for total and unique reads were obtained (R^2^ > 0.99). A boxplot of gene counts after TMM normalization revealed no bias in count distribution across samples (S1 Fig). Before performing the differential gene expression analysis, further multivariate analyses were carried out on the normalized gene count data to identify any discrepancies within and between individuals. The MDS plot, a technique aiming to detect meaningful underlying dimensions that allow explaining observed dissimilarities (distances) between samples, was also applied to the 24,596 genes after the TMM normalization. Inspection of the MDS plot showed Conv and LowI samples to partially overlap while a clearer distinction emerged for Conv and LowI *vs* Nutr (Fig 1).

In addition, sample BC7_Conv appeared to deviate markedly from the other samples. The interclass PCA also revealed that bull BC7_Conv laid outside the 95% confidence interval of its respective treatments (S2 Fig). However, interclass PCA did not identify any gene having extreme count profiles, which may have contributed to the transcriptome dispersion of animal BC7_Conv with respect to its treatment group. This sample was therefore retained for all the analyses. The total amount of expressed genes in liver was similar in all dietary treatments (Conv = 15,503, LowI = 14,994; Nutr = 15,576 genes). A total of 14,549 common genes were expressed in all groups. The correlation of mean gene expression levels between groups was very high (*r* = 0.92), suggesting that the major fraction of the liver transcriptome was not affected by nutrient ingestion.

#### Analysis of alternative splicing

We used the RNA-sequencing data to characterize and compare the patterns of alternative splicing in the 3 dietary groups. Multivariate Analysis of Transcript Splicing (rMATS) identified a total of 266 differential alternative splicing events: 14 mutually exclusive exons (MXE), 205 skipped exons (SE), 14 alternative 5′ splice sites (A5SS) and 32 retention introns (RI), while alternative 3’ splice sites (A3SS) events were absent. All 266 AS events were distributed across 238 multi-exon genes. When testing for differential alternative splicing in all comparisons, a total of 9 significant splicing events in 7 genes were found after multiple testing correction (Table 4, S3 Table). Interestingly, 2 SE in fibronectin 1 (*FN1*) (exon 33 and exon 25), 1 SE in leucine rich repeat (In FLII), interacting protein 1 (*LRRFIP1*) (exon 14), and 1 RI in complement component 2 (*C2*) were significantly associated with the Nutr diet in comparison with both LowI and Conv (Table 4).

**Table 4:** Significant differences in splicing events among the three diet groups (conventional, low-impact and nutraceutical).

GeneID	GeneSymbol	Comparison	Event Type	FDR	IncLevelDifference
ENSBTAG00000011424	*TPM2*	LowI vs Conv	MXE	0.0152	0.045
ENSBTAG00000008300	*FN1*	Nutr vs Conv	SE	0.0006	0.019
		Nutr vs Conv	SE	0.0204	0.024
		Nutr vs LowI	SE	0.0162	0.017
		Nutr vs LowI	SE	0.0162	0.022
ENSBTAG00000005354	*LRRFIP1*	Nutr vs Conv	SE	0.0031	0.113
		Nutr vs LowI	SE	0.0162	0.124
ENSBTAG00000007450	*C2*	Nutr vs Conv	RI	0.0272	0.030
		Nutr vs LowI	RI	0.0001	0.033
ENSBTAG00000006835	*MCAM*	Nutr vs LowI	SE	0.0006	0.284
ENSBTAG00000021319	*MFF*	Nutr vs LowI	SE	0.0180	-0.061
ENSBTAG00000014791	*CTH*	Nutr vs LowI	RI	0.0139	-0.202

LowI: low-impact diet; Conv: conventional diet; Nutr: nutraceutical diet

FDR: false discovery rate

MXE: mutually exclusive exon; SE: skipped exon; RI: retained intron; IncLevelDifference: difference in the exon inclusion level (%) between the experimental groups tested in each comparison

#### Identification of differentially expressed gene

RNA-sequencing data were used to study the effect of dietary composition on the liver transcriptome, with a focus on the identification of genes whose expression was significantly altered by LowI relative to Conv, Nutr relative to LowI, and Nutr relative to Conv. We identified 89 differentially expressed genes (DEGs), of which 52 were over-expressed and 37 were under-expressed in LowI compared with Conv. Comparing Nutr and LowI, we identified a total of 166 DEGs (79 up-regulated and 87 down-regulated). Regarding Nutr relative to Conv, 113 genes were differentially expressed (57 up-regulated and 56 down-regulated). Only three DEGs were commonly regulated among all 3 groups: nocturnin (*CCRN4L* or *NOCT)*, leucine rich adaptor protein 1-like (*LURAP1L)*, and pyruvate dehydrogenase kinase isozyme 4 (*PDK4)* (S3 Table).

#### Gene ontology and pathway analysis

We performed gene set enrichment analysis in order to identify over-represented gene ontology (GO) terms and Kyoto encyclopedia of genes and genomes (KEGG) pathways. In all comparisons, DEGs were strongly associated with lipid metabolism, molecular transport, and small molecule biochemistry. The full list of significantly enriched pathways is given in S4 Table. In addition, we characterized the transcription factors and microRNAs (miRNAs) that regulate DEGs (S5 Table).

The 89 DEGs found when comparing LowI with Conv were strongly associated with the isoprenoid biosynthetic process and glycogen metabolic process (S3 Table). In addition, the cholesterol biosynthesis canonical pathways and LXR/RXR activation were overrepresented (S4 Table). Upstream regulator analysis by Ingenuity Pathway Analysis (IPA) showed that the RAR-related orphan receptor C (*RORC*) gene, together with the insulin induced gene 1 (*INSIG1*), sterol regulatory element binding transcription factor 1 (*SREBF1)*, sterol regulatory element binding transcription factor 2 (*SREBF2*), and peroxisome proliferator-activated receptors alfa (*PPARA*) played a central role in mediating the cholesterol biosynthesis in animals fed with the LowI diet (S5 Table). In particular, these TFs affected the expression of genes involved in cholesterol biosynthesis (S5 Table). The 166 DEGs found when comparing Nutr with LowI treatments were related to metabolism of glycogen and carbohydrates, lipid homeostasis together with cholesterol biosynthesis and triglyceride metabolism, as well as fat cell differentiation and steroid biosynthetic process (S3 Table). The list of top upstream regulators revealed by IPA included *INSIG1* followed by the cooperatively transcriptional cofactors *RORC*, insulin receptor substrate (*IRS)* 2 and FA synthase (*FASN)* (S5 Table). TFs act in regulatory networks and can drive the expression of other TFs in a feed-forward and feedback manner. In fact, these TFs were in turn regulated by *SREBF1* and *SREBF2* together with other genes markedly down-regulated and mainly involved in cholesterol biosynthesis (S5 Table).

**Fig 2:**
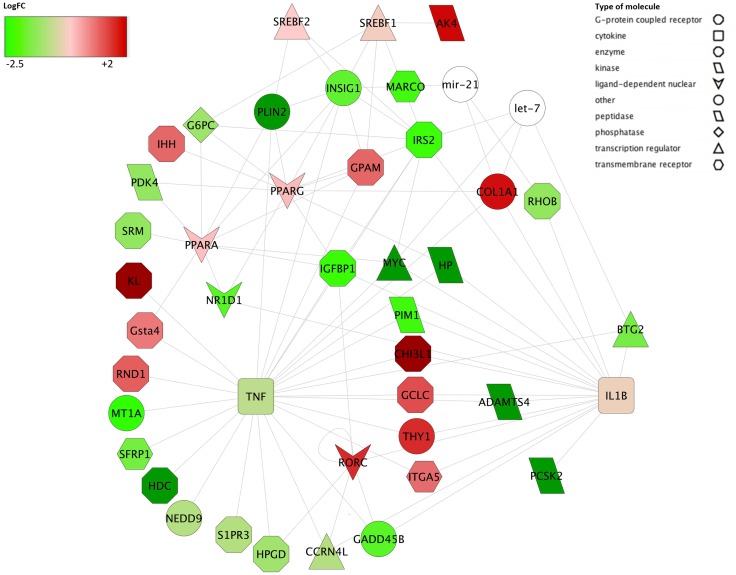
Regulatory network of transcription factors, miRNAs and target genes using Cytoscape. Schematic visualization of shared associations between transcription factors (TFs), miRNAs, and differentially expressed genes when comparing Nutr with Conv. The network is displayed graphically as nodes (genes, TFs, and miRNAs) and edges (the biological relationship between nodes). Node color intensity indicates the expression of the association: red = upregulation with Nutr diet, green = downregulation with Nutr diet. Node shape indicates whether it is a TF (triangle) or an miRNA (round) or other kinds of molecule.

When evaluating the effects of Nutr relative to Conv diet (113 DEGs), we observed that the most significantly enriched GO terms regarded the metabolism of energy reserve and differentiation of fat cell (S3 Table), including gluconeogenesis, triglyceride metabolic process, cholesterol biosynthesis and lipid homeostasis. Enrichment of the glutathione metabolism pathway was also found, as was activation of TR/RXR (S4 Table). Top upstream regulators included genes associated with the immune system, such as tumour necrosis factor (*TNF*) and the interleukin 1B (*IL1B*)(S5 Table; Fig 2), as well as TF playing important roles in lipid metabolism (i.e. the down-regulated *INSIG1*, *IRS2* and the up-regulated and *RORC* (Fig 2). In addition, Nutr down-regulated the expression of several target genes for *PPARA* compared to Conv diet (S5 Table).

### Correlations between differentially expressed genes and phenotypic traits

**Fig 3:**
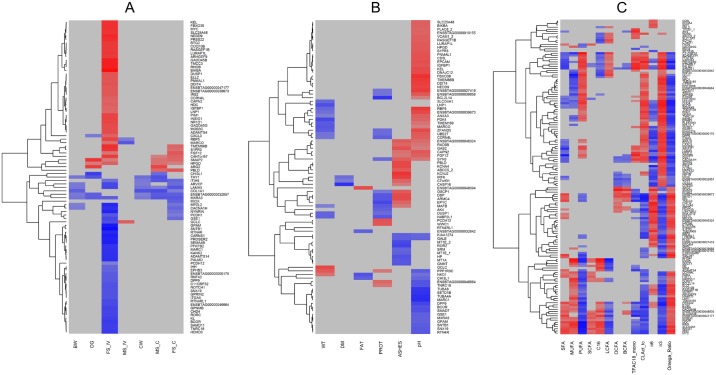
Heatmap of correlations between gene expression changes and phenotypic variations. Correlations were estimated for growth performances and carcass traits (A), liver weight, pH, and chemical composition (B), and liver fatty acid composition (groups of fatty acids) (C). The heatmap was generated using the CorrPlot package in R. The gene was included in the heatmap when a significant correlations for at least one phenotype was found (*P* < 0.05, Pearson product-moment correlations). Blue indicates negative correlations with values *r* < -0.51; grey indicates correlations values in the range -0.50 < *r* < 0.50; red color indicates positive correlations with *r* values > 0.51. BW: body weight; DG: daily gain; FS_IV: fat covering *in vivo*; MS_IV: body conformation; CW: carcass weight; MS_PM: carcass muscularity score; FS_PM: carcass fatness score; WT: liver weight. DM: dry matter; SFA: saturated fatty acids; MUFA: monounsaturated fatty acids; PUFA: polyunsaturated fatty acids; SCFA: fatty acids < C16; C16: fatty acids with 16 carbon chains; LCFA: fatty acids > C16; OCFA: odd-chain fatty acids; BCFA: branched-chain fatty acids; TFAC18_mono: Trans C18:1 fatty acids; CLAct_tc: conjugated linoleic acid *c*,*t/t*,*c* isomers; n_Ratio: ratio n-6/n-3.

Pearson correlations between DEGs and phenotypic traits are reported in Fig 3A–3C. Moderate to strong significant correlations (0.62 < *r* < 0.73) were found between DEGs and *in vivo* fatness scores (Fig 3A). Most of the DEGs positively correlated with the *in vivo* fatness scores were involved in the regulation of energy metabolic process (*IRS2)*, regulation of cholesterol biosynthesis *(INSIG1*), and circadian clock (e.g. *NOCT* and *NR1D1*). In contrast, the *RORC* and glycerol-3-phosphate acyltransferase mitochondrial (*GPAM*) genes, as well as the glycoprotein (transmembrane) Nmb (*GPNMB)*, which is associated with long-chain FA (LCFA) transport, were negatively correlated with fatness score (*r* = -0.732, -0.90 and -0.74, respectively). Additionally, a large number of DEGs correlated with pH in liver (Fig 3B). For instance, genes involved in the regulation of cation transmembrane transport [e.g. fibroblast growth factor 12 (*FGF12*) and kell blood group metallo-endopeptidase (*KEL)*] showed moderate positive correlations with liver pH (*r* = 0.62 and 0.71, respectively).

The association between liver FA composition and gene expression was calculated as a means of potentially identifying the association between the expression of lipid metabolism-related genes and FA contents in liver. Using the Pearson correlation approach it was possible to split liver FAs into two main clusters, which differed largely in their overall gene expression profiles. Gene cluster 1 contained 77 genes and had moderate to strong negative correlations with n-6 FA, n-6/n-3 ratio, FAs with 16 carbon chains, MUFAs, and SFAs, moderate to strong positive correlations with n-3 FA, CLA *c*,*t*/*t*,*c*, *trans* 18:1, as well as PUFAs and LCFAs, and no specific correlation with odd-chain FAs, branched-chain FAs and short-chain FAs (SCFAs) (Fig 3C). Gene cluster 2 contained 68 genes and had the opposite correlations. That is, we found CLA and n-3 to have moderate to strong correlations with genes related to the regulation of cholesterol biosynthesis (*GPAM*, *INSIG1*), glycogen biosynthetic process (protein phosphatase 1 regulatory (*PPP1R*) 3B, and *PPP1R3C*), circadian clock [e.g. aryl hydrocarbon receptor nuclear translocator-like (*ARNTL)*, *NOCT*, and *NR1D1*)], and regulation of FA metabolism (*INSIG1*, *IRS2*, and *PDK4*). On the other hand, SCFAs and FAs with 16 carbon chains showed moderate positive correlations with *INSIG1* (*r* = 0.71), *IRS2* (*r* = 0.54), and *FASN* (*r* = 0.67). In addition, n-6 FA was found to correlate with genes associated with carbohydrate catabolic process (e.g. glucose-6-phosphatase, catalytic subunit (*G6PC*), *r* = 0.6; myo-inositol oxygenase (*MIOX)*, *r* = -0.77), and the sphingolipid-mediated signaling pathway (sphingosine-1-phosphate receptor 3 (*S1PR3*), *r* = 0.67; spinster homolog 2 (*SPNS2*), *r* = -0.69). Interestingly, CLA, n-3, and n-6 FA also presented moderate to strong correlations with genes involved in inflammatory response, such as *LURAP1L* and insulin-like growth factor binding protein 1 (*IGFBP1)*, and with stress-response genes, such as *DDIT4* and *BTG* family member 2 (*BTG2*). Total PUFAs were highly associated with the expression of genes involved in LCFA transport (*GPNMB*; *IRS2* and peripilin 2 (*PLIN2*)).

Regarding the correlation of mineral contents with the expression profile of the DEGs, the highest number of significant and relevant correlations were found with Ca, Fe, Mn, Se and Zn (S6 Table). Significant and moderate to strong correlations were found for some minerals and genes involved in the cellular transport system: e.g., Mn and Zn were negatively correlated with ATP-Binding Cassette, sub-family C member 5 (*ABCC5*) (*r* = -0.70 and -0.83, respectively), and Ca and Se were negatively correlated with ATP-Binding Cassette, Sub-Family G Member 8 (*ABCG8*) (*r* = -0.62 and -0.77, respectively). Positive moderate correlations were found for Zn contents and metallothionein (*MT*) *1A* (*r* = 0.64), *1E1* (*r* = 0.78) and *1E2* (*r* = 0.68).

## Discussion

In the present experiment, we evaluated the effects of feeding strategies designed to have the least interference with growth performances but differing in the supply of specific nutrients in young Belgian Blue × Holstein bulls. As expected, the dietary changes had small or no relevant influence on growth performances and main carcass traits, and on the composition of major nutrients in the liver, but they had a major influence on liver FA profile and clearly affected the transcriptome expression of bovine liver.

### Dietary treatments and phenotypic traits

The use of the LowI diet, where extruded full-fat soybeans replaced the soybean meal of the Conv diet, notably decreased protein and increased n-6 FA intakes. Inadequate protein intake may impair growth and host immunity with particularly detrimental effects on the T cell system, resulting in an increased incidence of infections [22], and may also affect the insulin/glucagon ratio [23] and the rate of hepatic glycogen synthesis [24]. However, an excess of protein can also be detrimental to ruminant health, as an excess of rumen-degradable N increases lameness and laminitis, and certain types of proteins may cause allergic histaminic reactions [25]. The effect of including extruded full-fat soybean rich in 18:2*c9*,*c12* in the diet was evident primarily in an increase in the proportion of 18:2*c9*,*c12* in the liver fat. Other experiments conducted on a larger number of bulls found that increased amounts of extruded full-fat soybeans increased the 18:2*c9*,*c12*, 18:2*c9*,*t11*, 18:1*t11*, or other intermediates of rumen biohydrogenation, in intramuscular, intermuscular and subcutaneous fat in beef [25–26].

Notably, Nutr increased n-3 FAs, CLA, and vitamin E intakes compared with LowI, due to the inclusion of linseed CLA, and vitamin E. This feeding treatment had no effect on growth performances, except for a reduction in the *in vivo* subcutaneous fat covering. Data on the effects of CLA on body fat conflict, as the reduction of body fat was not always confirmed in growing ruminants [25–26, 27]. Notable alteration to the FA profile of liver fat was instead induced without apparently affecting the liver fat content. In particular, linseed, rich in 18:3*c9*,*c12*,*c15*, caused a marked increase in this FA in the liver fat. In general, the liver fat of bulls fed Nutr had higher proportions of LCFA, n-3 FA, and CLA isomers (2.5-fold) compared with LowI. Compared with Conv, Nutr and LowI caused a significant increase in the liver content of rumen hydrogenation intermediates, such as *trans-*18:1 FA and CLA, which were found to inhibit *de novo* FA synthesis in the mammary gland [28].

Concerning mineral concentrations in liver, we observed that most of the trace and macro-mineral contents of cattle liver in our study were in line with data from the literature [29–31]. Changes in the dietary content of macro-elements in the LowI and Nutr diets only nominally affected the liver contents compared with Conv. A larger number of animals would be required to draw statistical conclusions from these differences.

### Effects of reduced protein and mineral supplementation, and increased soybean oil supplementation on bovine liver transcriptome

The changes in the protein and lipid profiles characterizing LowI diet were associated with down-regulation of glycogenesis and up-regulation of cholesterol biosynthesis in bovine liver. Liver metabolism can be regulated by various metabolites, e.g. NEFAs and glucose [32]. The LowI diet seemed to affect the regulation of insulin homeostasis, as suggested by the over-expression of insulin target genes, such as *FASN* and *PDK4* (S5 Table). Coupled to that, up-regulation of target genes for *SREBF1*, *SREBF2* and *PPARA* supported the hypothesis that LowI enhance regulation of cholesterol biosynthesis in bovine liver. The activation of *RORC* could also play a role on the induction of cholesterol biosynthesis, the inhibition of glycogen synthesis and the regulation of the circadian clock. Circadian clock controls energy metabolism through several genes involved in FA, cholesterol and glucose metabolism in liver, which are regulated in circadian or diurnal patterns [33]. However, our results did not allow ascertaining if the ingestion of higher amounts of PUFA caused the increase in cholesterol biosynthesis and consequently, an enhanced biliary cholesterol secretion [34]. Besides to the aforementioned changes in energy metabolism, we observed the activation of genes involved in immune response which may be explained by the lower intake of protein and/or the higher intake of dietary n-6 PUFAs (linoleic or arachidonic acid) which alter cell membrane fluidity and indirectly affect inflammatory processes in that they increase the pro-inflammatory response [35].

Over the past decade, miRNAs have emerged as novel elements in the rapid, reversible regulation of transcription and translation [36]. MiRNA are small, non-coding RNA molecules (~19–24 bp in length) that are synthesized from short hairpin precursors and that reportedly degrade or inhibit translation of their target genes by binding to the 3′ untranslated region (UTR) of coding mRNA [36]. Interestingly, the LowI diet modified the expression of two mir-185 target genes, which are involved in cholesterol biosynthesis (S5 Table), suggesting that this miRNA may also play a role in liver lipid metabolism along with the above-mentioned TFs. Indeed, miR-185 is a key micro-RNA identified as a regulator of *de novo* cholesterol biosynthesis and low-density lipoprotein uptake; inhibition of miR-185 resulted in decreased *SREBP-2*-dependent gene expression, LDL uptake, and HMG-CoA reductase activity [37].

### Effects of n-3, CLA and vitamin E supplementation on bovine liver transcriptome

A large degree of transcriptomic adaptation to the n-3 and CLA FAs was observed in the liver of bulls fed the Nutr diet. Biological function analysis revealed under-expression of TFs regulating lipid metabolism, such as *INSIG1* and *IRS2* (Fig 2). In addition, *FASN* and the target genes for *SREBF1* and *SREBF2* were strongly inhibited by the Nutr diet (Fig 2). When cells have sufficient sterol levels, *INSIG1* retains the *SREBP1* cleavage-activating protein *(SCAP)-SREBP1* in the endoplasmic reticulum and consequently inhibits *SREBP1*-mediated gene expression [38]. In addition, *INSIG1* is itself a target of *SREBP*, and is repressed when *SREBPs* are inactive [39]. In bovine mammary epithelial cells, down-regulation of *INSIG1* and *FASN*, a key enzyme involved in *de novo* FA synthesis by oleic acid, linoleic acid, VA and alpha-linolenic acid (n-3), was observed [40–41]. Hiller et al. [42] also found evidence that supplementation with n-3 FA caused reduced expression of various genes involved in FA synthesis, including *FASN*, in Holstein cow liver. In light of the above observations, it is tempting to speculate that PUFAs (in particular CLA and n-3 FA) reaching the liver affect the expression of both *SREBP1* and *INSIG1* to inhibit *SREBP1*-mediated gene expression, and consequently, at least partially, likely reduce the rate of lipogenesis occurring in bovine liver in a negative feedback mechanism. In addition, the lower expression of *IRS2* may support an increase in the extent of FA oxidation [43] in Nutr, compared with other diets. With Nutr, the increased amount of PUFAs reaching the liver likely increased hepatic oxidation of FA, driving liver metabolism towards lipid uptake and utilization, unlike what was observed in bulls fed LowI. This hypothesis is further supported by the down-regulation of *PDK4* (S3 Table). Depletion of *PDK4* increases PDC activity, which allows pyruvate to be channeled to the tricarboxylic acid cycle for complete oxidation and as a result pyruvate is not available for gluconeogenesis [44]. It would seem that under-expression of *PDK4* came about by an increased amount of PUFAs reaching the liver after administration of Nutr, consistently with previous studies [45]. Additionally, down-regulation of the key gluconeogenic factor G6PC seems to be consistent with a negative effect on gluconeogenesis in liver. Considering the effects of Nutr on genes related to immune/stress response, our results seemed to support the hypothesis that supplementation with n-3 FA and CLA may decrease the production of inflammatory eicosanoids, cytokines, and ROS, and also decrease *de novo* FA synthesis in liver, as published elsewhere [46]. This could explain why CLA supplementation may improve the health status of ruminants [21].

Several target genes for the miR Let-7 and Let-7a-5p were down-regulated in the animals on the Nutr diet (S5 Table), supporting previous evidence concerning the role of the Let-7 family of miRNA in regulating glucose metabolism in the liver [47]. Target genes for miR-21 were differentially expressed in Nutr vs LowI (6), and Nutr vs Conv (3) (S5 Table), according to recent findings concerning its putative effect on the regulation of triglyceride and cholesterol metabolism [48].

The role of dietary nutrients, such as carbohydrates, FAs, and protein, in regulating alternative splicing events and in controlling nutrient metabolism through variations in the splicing of the pre-mRNA encoding enzymes involved in various metabolic pathways has been recently highlighted. For instance, splicing of the pre-mRNA encoding *G6PD* is modulated by carbohydrates and FA [49]. Interestingly, we found an SE event in *PFKFB2*, a gene involved in the control of glycolysis, which was also upregulated in the Nutr group (S3 Table). However, the differences in the inclusion levels of this splicing event between our experimental groups were not significant (data not shown). On the other hand, in the Nutr group we found a significant difference in the percentage of inclusion of AS events in genes involved in the innate immune response, such as *FN1*, *LRRFIP1* and *C2* [50–52]. The possibility that these events may be induced in the individuals on the Nutr diet, favoring alternatively spliced isoforms with specific roles, cannot be excluded. Interestingly, in recent years multiple roles of alternatively spliced FN isoforms have been identified in humans [53].

### Correlations between gene expression changes and phenotypic variations

The highest number of significant and relevant correlations with DEGs was found with those traits that were significantly different in at least one comparison between diets (Fig 3A–3C). Interestingly, fat covering *in vivo* showed a moderate positive correlation with *LURAP1L* (*r* = 0.79), which was one of the three genes differentially expressed in all comparisons. Its paralog, *LURAP1*, was shown to play a crucial role in obesity-induced inflammation [54]. As expected, moderate to strong correlations were found between fat covering *in vivo* and genes related to energy reserve metabolic process. A moderate positive correlation was also found between fat covering *in vivo* and *NR1D1* (*r* = 0.73), which has a role in the network of Fig 2, further confirming the link between energy metabolism and the expression of clock genes [55].

Genes involved in membrane transport showed moderate positive correlations with liver pH, providing support for a connection between hepatic membrane transport and pH regulation mechanisms [56].

Finally, the results of correlation analyses of liver FA profiles revealed that CLA and n-3 FA might exert similar effects on bovine liver fat metabolism (increased FA oxidation and decreased lipid synthesis), as they seemed to act on lipid-related genes (e.g., key TFs, such as *INSIG1* and *IRS2* together with some of their target genes) with likely equivalent effects (same degree and direction of correlations) (Fig 3C). On the other hand, it is interesting to note that, when looking at the correlation values of the DEGs involved in the inflammatory/stress response, such as *LURAP1L*, *BTG2*, and *IGFBP1*, CLA and n-3 FA seemed to have the opposite effect to that of n-6 FA (negative *vs* positive correlations), thus confirming the anti-inflammatory properties of the former and the pro-inflammatory potential of dietary n-6 PUFA [46].

In general, weak correlations between DEG and mineral contents were found (S6 Table), suggesting that the variation in the dietary mineral content probably did not have a great effect on the bovine liver gene expression profile. Interestingly, moderate positive correlations were found between *MT1A* and *MTE1* and liver Zn content, confirming that cells are likely to protect themselves from zinc toxicity by inducing MTs, which bind it tightly by sequestering it in organelles, or by exporting it [57].

## Conclusions

In summary, this trial suggested the feasibility of using low environmental impact feeding regimes (low protein and mineral contents) in livestock production without compromising animal performances and carcass characteristics. The RNA-sequencing data here obtained have broadened our understanding of the effects of variations in feeding strategies on bovine liver transcriptome, and have unraveled, at least partially, the mechanisms underlying the relationships between nutrients and phenotypic variations. In particular, the balance between glucose and lipid metabolism was affected by both low-impact diets (LowI and Nutr), while the greatest effects on liver transcriptome resulted from the addition of linseed and CLA (Nutr). *INSIG1*, *IRS2* and *RORC*, key TFs controlling the expression of lipid-related genes, clearly played a pivotal role in mediating the observed effects on gene expression. On the other hand, the changes in mineral composition seemed not to considerably affect bovine liver gene expression profile. The increased supplementation with vitE occurring in Nutr could likely be related to the regulation of glutathione metabolism.

Thereby, our results helped in clarifying the role of bovine liver in the biological response to specific nutrients variations. The regulation of liver metabolic processes involved in lipid metabolism may subsequently influence fatty acid deposition into the fat of ruminant products. It is clear however, that validation on a larger set of biological samples is needed in order to confirm the present RNA-sequencing findings and draw more robust conclusions about the interactions between diet and bovine liver metabolism.

## Material and Methods

### Animals, housing and diets

This study was part of a large experiment aimed at investigating the effects of different feeding strategies on animal performances, carcass traits, and chemical and FA compositions of different tissues (muscle, liver, and adipose tissue) in 48 crossbred heifers and young bulls (15–19 months old at slaughter). The project was approved by the Ethical Committee for the Care and Use of Experimental Animals of the University of Padua (CEASA) and all methods were carried out in accordance with the approved guidelines. Following the experimental protocol for animal care, health status was monitored daily by a technician and three times per week by a veterinarian.

Briefly, 24 Belgian Blue × Brown Swiss (12 ♂ and 12 ♀), and 24 Belgian Blue × Holstein (12 ♂ and 12 ♀) animals were weighed and housed in 12 pens (4 animals per pen), balanced by sex, breed, age and initial BW, and considering the representativeness of half-sib families based on pedigree information. The animals were assigned to 1 of 3 dietary treatments following a factorial arrangement. All animals belonging to the same pen were fed the same dietary treatment.

After an adaptation period, a first group (Conv) was fed a conventional diet containing 14.4% crude protein (CP) on a DM basis, mainly from soybean meal, and supplementation of minerals and vitamins for the whole experimental period of 9 months (Table 5). The second group was fed for the whole experiment a LowI diet containing 10.7% CP on a DM basis, obtained by substituting all the soybean meal with a small amount of full-fat soybean, and reducing the mineral supplementation (Table 5).

**Table 5:** Ingredient and chemical composition of the diets.

	Conventional diet (Conv)	Low impact diet (LowI)	Nutraceutical diet^1^ (Nutr)
**Ingredient composition, kg/d:**			
Corn silage	2.53	2.53	2.53
Corn meal	3.61	3.61	3.61
Wheat bran	0.62	0.62	0.62
Wheat straw	0.65	0.65	0.65
Sugar beet pulp	1.06	1.06	1.06
Grape-seed meal	0.18	0.18	0.18
Soybean meal (44% CP)	1.29	-	0.20
Corn meal	-	0.79	0.53
Extruded soybeans, full fat	-	0.48	-
Linseed, full fat	-	-	0.48
Vitamins-minerals	0.20	0.20	0.20
**Chemical composition:**			
Dry matter, g/kg as fed	757	755	757
Crude protein, g/kg DM	144	107	107
Neutral detergent fiber, g/kg DM	300	295	296
Starch, g/kg DM	360	418	401
Ether extract, g/kg DM	30	42	55
Ash, g/kg DM	44	39	41
Vitamin A	4123	4207	4176
Vitamin E	25	30	116
**Net energy content, UFC/kg DM**	1.02	1.03	1.04

^1^The Nutraceutical diet was also top dressed with 80 g/d of a commercial rumen protected conjugated linoleic acid product (SILA, Noale, VE) providing 5.57 g/d of *cis-9*, *trans-11* CLA and 5.41 g/d of *trans-10*, *cis-12* CLA. All diets contained 0.79 g/kg dry matter of Na, and 401 IU/kg dry matter of Vitamin D3. CP: crude protein; DM: dry matter.

In the third treatment, the animals were fed the LowI diet for the first 5 months, and the Nutr diet for the following 4 months (for young bulls). In the Nutr diet, the full-fat soybeans in the LowI diet were replaced with linseed rich in n-3 FA, adding a high level of vitamin E [as an antioxidant to compensate for a possible reduction in beef shelf-life due to increased oxidation of the PUFAs derived from linseed], and top dressed with 80 g/d of a commercial CLA mixture (SILA, Noale, Italy). Whole linseed was chosen because this oilseed is rich in n-3 FA (54.2% alpha-linolenic acid), and its seed coat may protect PUFA from rumen biohydrogenation and increase the passage of PUFA to the duodenum. The diets were distributed once a day at 8.00 a.m., and the animals had free access to the manger and drinkers. The amount of each feed ingredient loaded into the mixer-wagon and the weight of the mixture uploaded into the manger of each pen were recorded daily. The orts remaining in the mangers were weighed and sampled by pen weekly.

Animal BW and ADG were recorded for each bull 3 times during the experiment. An operator licensed for carcass evaluation according to the SEUROP grading system (European Commission, 1991) made monthly evaluation of the *in vivo* body condition of each bull as described by Schiavon et al. [21]. Conformation was linearly scored from S+ (all muscle profiles extremely convex; superior muscle development) to P- (all muscle profiles concave to very concave; very poor muscle development). Conformation was expressed in numerical terms: S+ = 6.33; S = 6; S- = 5.66; …, P+ = 1.33; P = 1.00; P- = 0.66. Fat covering was scored linearly from 1 (very lean) to 5 (very fat) using a combined visual and palpation approach, taking into account the presence and thickness of subcutaneous fat depots at specific points of the body.

### Slaughtering, carcass traits, and sample collection

Animals were transported to the slaughterhouse after 280 days on experimental feed with an average target slaughtering weight of approximately 670 kg. They were all fasted for 1 day and slaughtered all together at 532 ± 36 days old. The animals were killed using captive bolt pistol in the stunning process and all efforts were made to minimize suffering. Carcasses were individually weighed and scored for conformation and fat covering according to the SEUROP system (European Commission, 1991). Dressing percentage was computed as the ratio between the carcass weight 24 h after slaughter and BW.

Immediately after slaughter, liver tissue samples were collected from all the animals, weighed, then stored at -20° for chemical and FA analyses. The liver tissue samples for RNA-sequencing analyses (~30 mg) were placed in cryogenic, sterile, DNAase- and RNAase-free vials (Cryo.s, Greiner Bio One, Frickenhausen, Germany) and immediately snap-frozen in liquid nitrogen and stored at -80°C until RNA extraction. Since sex and breed differences in liver transcriptome have been reported for several species, including cattle [58], only the 12 males from the same crossbreed (Belgian Blue × Holstein) were selected for subsequent analyses. Female animals were excluded because of the lower importance of heifers for meat production and as hormonal fluctuations might affect liver gene expression profiles in mammals [59]. Belgian Blue × Holstein crossbreed young bulls were instead chosen respect to Belgian Blue × Brown Swiss crossbreds because Holstein breed is much more used than Brown Swiss in dairy farms worldwide and as they were more balanced in terms of half-sib families. Data for the transcriptome profiling therefore came from 4 Belgian Blue × Holstein bulls for the Conv diet, 4 for LowI, and 4 for Nutr. One bull on the Conv diet was removed from the study due to health problems unrelated to the dietary treatment.

### Chemical analysis

#### Liver proximate composition

The 50 g liver samples were sliced, ground and homogenized using a food processor (Grindomix GM200; Retsch, Haan, Düsseldorf, Germany), and subsamples of 20 g were used to determine their chemical composition according to AOAC (2000). In brief, moisture was determined after drying at 102°C for 16 h, while ash was determined after mineralization at 525°C. Total lipids were analyzed by extraction with petroleum ether, and protein was estimated by difference, in accordance with Schiavon and colleagues [25]. Liver pH was measured on fresh samples using a Delta Ohm HI-8314 pH-meter (Delta Ohm, Padova, Italy).

#### Liver fatty acid profile

The left lobe of each liver was sliced, ground, mixed, and then homogenized for 10 sec at 4500 g (Grindomix GM200; Retsch, Haan, Düsseldorf, Germany). Two subsamples of 50 to 60 g each were used to determine the FA composition. Samples were stored at -20°C until analysis. After conservation, a subsample (2.00 ± 0.005 g of liver, approximately 44 mg of fat) was freeze-dried with a CoolSafe 90–80 freeze dryer (Scanvac, Stockholm, Sweden), according to the manufacturer’s instructions. Freeze-dried samples were methylated as described by Jenkins [60] to limit trans-isomerization of the FAs [61]. The samples obtained were analyzed for their FA profiles using a GC × GC instrument (7890A; Agilent Technologies, Santa Clara, CA, USA) with two columns in series and equipped with a modulator (G3486A CFT; Agilent Technologies), an automatic sampler (7693A; Agilent Technologies) and a flame ionization detector (FID) connected to the Agilent Chemstation chromatography software (Agilent Technologies). Operating conditions and GC reference standards were previously reported in detail [27]. The liver FA composition was expressed as grams per 100 g of total FA.

#### Liver mineral profile

Liver samples were digested using the microwave digestion method (Milestone Start D, Milestone Srl, Sorisole, BG, Italy) (AOAC, 2000). Samples of approximately 1.0 g were digested with 7 ml of HNO_3_ and 2 ml of H_2_O_2_ 30% in the microwave digestion system. The samples and acid mixture were placed in suitable inert polymeric microwave vessels. Each vessel was sealed and heated in the microwave digestion system. The temperature program was as follows: 15 min to reach 200°C and held for 18 min. The resulting solution was cooled to 35°C and diluted to 25 ml with distilled water. Determination of 27 micro- and macro-elements in this clear solution was carried out by SPECTRO ARCOS ICP-OES (SPECTRO Analytical Instruments, GmbH, Kleve, Germany). Instrument operating parameters were optimized for aqueous solutions with undissolved organic material. Calibration standards were matched with 1% absolute ethanol (Prolabo VWR International PBI srl, Milan, Italy). The concentration ranges of the calibration solutions were 0–250 mg/L for macro-elements and 0–20 mg/L for micro-elements. The elements to be determined were added from single element solutions (Inorganic Ventures, Christiansburg, VA, USA). The measured and certified values of all elements were in excellent agreement. Element contents were expressed as mg/kg fresh tissue.

### Statistical analyses of phenotypic data

A Kruskal Wallis non-parametric test with Bonferroni correction was used to identify differences in the phenotypic traits among the 3 dietary treatments (Conv, LowI, Nutr). The level of significance was set at an adjusted *P-*value < 0.05.

### Liver transcriptome profiling

#### RNA extraction

Total RNA was isolated from liver samples (30 mg) stored at -80°C using the RNAeasy Mini kit (Qiagen, Hilden, Germany), according to manufacturer’s instructions. DNAse treatment (Qiagen, Hilden, Germany) was carried out to avoid possible contamination from genomic DNA. RNA purity and concentration were determined using a NanoDrop ND-1000 spectrophotometer (Thermo Scientific, Wilmington, DE) and RNA integrity was assessed using the Agilent 2100 Bioanalyzer (Agilent Technologies, USA). All samples had an RNA integrity number above 8.

#### Library preparation and RNA-sequencing

Eleven tagged libraries for RNA sequencing experiments were prepared from extracted mRNA using Agilent's SureSelect Strand Specific RNA Library Preparation Kit (Agilent Technologies), following the manufacture’s instruction. Briefly, poly(A) mRNA was purified from 200 ng of total extracted RNA and fragmented using an RNA-seq Fragmentation Mix. First-strand and second-strand cDNA were synthetized and end repaired. Adenylation of cDNA 3’ ends and adaptor ligation were performed. Sixteen cycles of PCR amplification were used to amplify and index the adaptor-ligated cDNA library, and the PCR products were purified and size selected using the SPRIselect reagent kit (Beckman Coulter, Brea, CA, USA). A Qubit RNA Assay kit in a Qubit 2.0 Fluorometer (Life Technologies, CA, USA) was used to measure library concentrations. Individual libraries were monitored for insert size using the Agilent High Sensitivity DNA assay on the Agilent Bioanalyzer 2100 system (Agilent Technologies, Palo Alto, CA). Multiplexed paired-end sequencing 2 × 100 bp was carried out on an Illumina Hi-Seq 2500 (UC Davis Genome Center, Davis, CA, USA). To this end, a total of 3 lanes were run. In order to avoid potential biases related to divergent sequencing qualities, four barcoded libraries were loaded in each lane by randomizing samples belonging to the three diets. Raw Illumina sequencing data have been deposited in GenBank (SRA) (PRJNA303144, BioProject SRP066556, study accession).

#### Reads processing and quality control

Initial quality control was carried out with the FastaQC software (http://www.bioinformatics.babraham.ac.uk/projects/fastqc/) version 0.11.4 [62]. Raw reads were then trimmed for low quality bases using CLC Genomics Workbench v 8.5 as follows: 1) all reads were trimmed of 10 nucleotides at the 5’ end; 2) Illumina adapters were removed; 3) reads with >5% nucleotide with PHRED scores <20 were filtered out.

#### Reads mapping

The trimmed reads were mapped to the *Bos taurus* reference genome (UMD3.1, release 81.3) using CLC Genomics Workbench v 8.5 (CLC Bio, Aarhus, Denmark) with default parameters, except for a length fraction of 0.8 and a similarity fraction of 0.8. The alignments were saved as BAM files. In order to validate the steadiness of counts predicted by the CLC Genomics Workbench, gene count estimation was also performed using the HTSeq-count tool (part of the HTSeq framework, version 0.7.1, in ‘union’ mode), an open source toolkit that allows the input of raw counts from aligned reads to be annotated with gene names based on genomic features [63]. To compare the two methods, Pearson and non-parametric Spearman rank correlations were applied using the rcorr function in the Hmisc package in the R environment (v 3.1).

#### Identification of alternative splicing events

In order to improve the alternative splicing analysis, instead of using the previously obtained alignments, trimmed reads were mapped against the *Bos taurus* Ensembl genome UMD3.1 release 81.3 in a splicing-aware manner using STAR aligner v2.4.1d with the following arguments “—outSAMtype BAM SortedByCoordinate—outSAMmapqUnique 60—outFilterMultimapNmax 2—outFilterMismatchNmax 10—alignEndsType EndToEnd” [64]. Alternative 3′ (A3SS) and 5′ splice sites (A5SS), skipped exons (SE), mutually exclusive exons (MXE), and retained introns (RI) from the alignments were quantified using rMATS v3.2.0.beta [65] with Ensembl cow UMD3.1 annotation release 81.3. Fragment insert size and standard deviations, required to run rMATS, were calculated for each sample using the Picard CollectionInsertSizeMetric tool. Alternative splicing (AS) events were detected using both junction counts and reads on target. To identify different putative AS events due to the three different diets, rMATS evaluated whether differences in the exon or intron inclusion levels (IncLevelDifference) of an AS event between two conditions exceeds a false discovery rate (FDR) threshold of 5%.

#### Identification of the differentially expressed genes

The read counts obtained from the CLC Genomics Workbench were used to estimate exon expression and identify DEGs, which was done using the Bioconductor package edgeR version 3.10.0 [66] in the R environment (version 3.1). The “*calcNormFactors*” normalization function of the edgeR package was used to find a set of scaling factors for the library sizes that minimized the log-fold changes between samples. The scale factors were computed using a trimmed mean of M-values (TMM) between samples [66]. Principal component analysis (PCA) and Multidimensional Scaling analysis (MDS) were performed with FactoMineR and Ade4 packages (version 1.23) to assess whether any particular array made a large contribution to the variability in the gene expression data, that is, whether it retained most of the information. The differences that emerged from the PCA analysis were assessed using the Monte Carlo Permutation Procedure (999 replicates; “*randtest*” function) of the Ade4 package in R [67]. DEGs were detected by applying a generalized linear model (GLM) likelihood ratio test, which fits negative binomial (NB) with the Cox-Reid dispersion estimates [67]. Common, trended and tagwise dispersions were calculated using the “*estimateDisp*” functions in the edgeR package. The DEGs were then determined using the GLM likelihood ratio test then each of the treatment groups was compared using the contrast argument of the “*glmLRT*” function in the edgeR package. EdgeR generated a list of genes with fold changes (FC), *P*-values, and the associated Benjamin-Hochberg FDR values. A further filtering step was introduced to delete transcripts with low expression levels (log2 < 4 in 90% of samples). The false discovery rate was then recalculated on the basis of the final transcript number, by implementing in Rstudio the FDR calculation method described by Storey and Tibshirani [68]. Only transcripts with FDR < 0.05 were retained for subsequent analyses. Visualization of the DEGs found to be commonly regulated in the three diet groups was performed using Venny, an interactive tool available at http://bioinfogp.cnb.csic.es/tools/venny/.

#### Functional analysis of differentially expressed genes

To gain a better understanding of the functional implications of these DEGs among the different dietary treatments, GO [69] and KEGG [70] enrichment analyses were performed using Cytoscape V2.7 (http://cytoscape.org/) with the ClueGo V1.3 plug-in 3 [71]. A single cluster analysis was used for each comparison. ClueGO determines the distribution of the gene list for the various GO terms and pathways. The *P-*value was calculated using right-sided hypergeometric tests and the Benjamini-Hochberg correction for multiple testing (FDR <0.05). Together with this stringent FDR threshold, a high kappa value (0.4) enabled us to precisely select enriched GO terms in highly connected genes. The size of the nodes reflects the degree of enrichment of the terms. The network was automatically laid out using the organic layout algorithm in Cytoscape. Functional groups were created by iterative merging of the initially defined groups, according to the predefined kappa threshold. Only functional groups represented by their most significant term were visualized in the network [71]. A complementary approach, which involved feeding the list of selected genes into an IPA (ver. 5.5, Ingenuity Systems, Redwood City, CA), was also taken to identify relevant categories of molecular functions, cellular components and biological processes. Using this approach, we identified statistically overrepresented functional GO annotations, and determined their up- or down-expression and group-specific transcriptional networks. All listed or reconstructed cellular pathways were derived from the expert annotated database provided by the Ingenuity Knowledge Base. The IPA annotations follow the GO annotation principle, but are based on a proprietary knowledge database of over 10^6^ protein-protein interactions. The IPA output included biological functions and signaling pathways with statistical assessment of the significance of their representation based on Fisher’s Exact test. IPA computed the networks and ranked them according to statistical likelihood [72]. Only the canonical pathways that presented a log2 (*P*-value) exceeding 1.30 (FDR < 0.05) were considered.

### Correlation analyses between differentially expressed genes and phenotypic traits

To find possible associations between the set of the previously identified DEG and phenotypic traits, the log2 of normalized gene counts for all DEGs expressed among the three dietary treatments were correlated with: i) animal growth performance, ii) carcass traits, iii) liver weight and chemical composition, iv) liver mineral composition, and v) liver FA composition. Pearson product-moment correlation coefficients and *P-*values were calculated using the Hmisc package (3.17.2) in the R environment. The level of significance was set at *P* < 0.05. Heatmaps and dendograms were generated using the significant correlations for at least one trait by the gplots package in implemented in the R environment (v 3.1).

## Supporting Information

S1 Fig Boxplot of the natural log transformed estimated gene counts per animal after trimmed mean of M-values (TMM) normalization. Boxplots were created with the ggplot2 package in the R statistical environment.
(TIFF)

S2 Fig Interclass principal component analysis (PCA) of normalized gene count data. The plot was created using principal component analysis function implemented in the ade4 R package with samples as variables and differential expression comparison groups as class levels.
(TIFF)

S1 Table Mapping summary statistics of RNA sequencing data. Comparative results obtained by CLC Genomics Workbench and HTSeq-count tools.
(XLS)

S2 Table Differential alternative splicing events for all comparisons using rMATS. Significant differences (FDR < 0.05) in the inclusion of splicing events across the experimental diets. Conv: conventional diet; LowI: low-impact diet.
(XLSX)

S3 Table Differentially expressed genes (FDR < 0.05) for comparison among experimental diets. LowI: low-impact; Conv: conventional diet; Nutr: nutraceutical diet.
(XLSX)

S4 Table Canonical pathways enriched in the experimental diets using IPA. The ratio values (number of molecules in a given pathway that meets cut criteria, divided by total number of molecules that make up that pathway) are also presented.
(XLSX)

S5 Table Upstream regulators analysis results for comparison among experimental diets using IPA. IPA prediction of upstream regulators that are activated or inhibited to explain the upregulated and downregulated genes observed in the experimental diets.
(XLSX)

S6 Table Correlation matrix of Pearson product-moment correlations between liver mineral profiles and differentially expressed genes. Pearson product-moment correlation coefficients and *P* values are reported for all correlations.
(TXT)
