# Supplementary figures and images for: Transcriptomic Changes in Liver of Young Bulls Caused by Diets Low in Mineral and Protein Contents and Supplemented with n-3 Fatty Acids and Conjugated Linoleic Acid
Source: PLoS One. 2016 Dec 8;11(12):e0167747. doi: 10.1371/journal.pone.0167747 (PMC5145186; doi:10.1371/journal.pone.0167747)

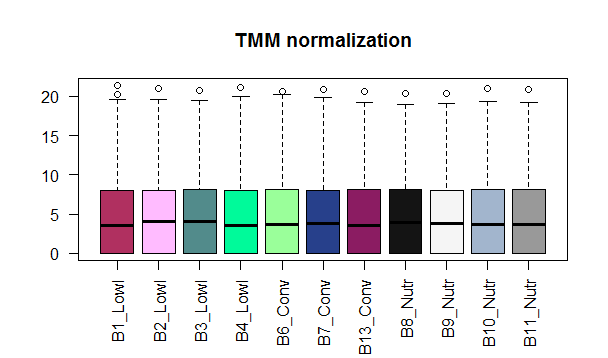

Supplement: S1 Fig — Boxplots were created with the ggplot2 package in the R statistical environment. (TIFF) [file pone.0167747.s001.tiff]

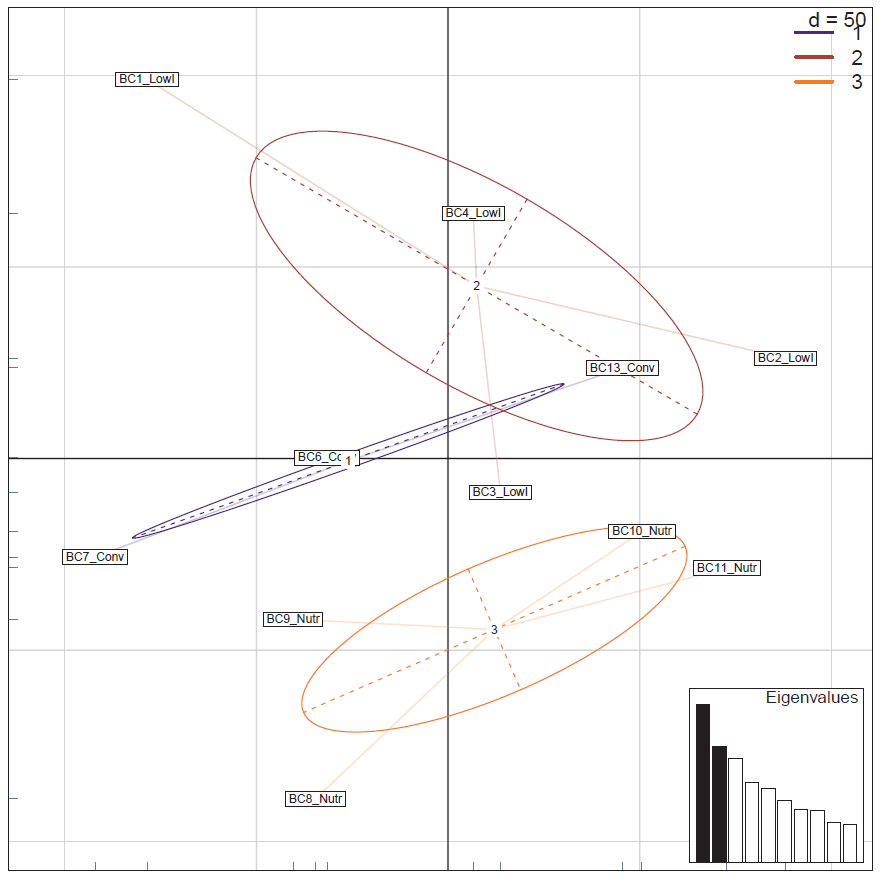

Supplement: S2 Fig — The plot was created using principal component analysis function implemented in the ade4 R package with samples as variables and differential expression comparison groups as class levels. (TIFF) [file pone.0167747.s002.tiff]
